# Comparative High-Resolution Mapping of the Wax Inhibitors *Iw1* and *Iw2* in Hexaploid Wheat

**DOI:** 10.1371/journal.pone.0084691

**Published:** 2013-12-23

**Authors:** Haibin Wu, Jinxia Qin, Jun Han, Xiaojie Zhao, Shuhong Ouyang, Yong Liang, Dong Zhang, Zhenzhong Wang, Qiuhong Wu, Jingzhong Xie, Yu Cui, Huiru Peng, Qixin Sun, Zhiyong Liu

**Affiliations:** 1 State Key Laboratory for Agrobiotechnology / Beijing Key Laboratory of Crop Genetic Improvement / Key Laboratory of Crop Heterosis Research & Utilization, Department of Plant Genetics & Breeding, China Agricultural University, Beijing, China; 2 Plant Science and Technology College, Beijing University of Agriculture, Beijing, China; Nanjing Agricultural University, China

## Abstract

The wax (glaucousness) on wheat leaves and stems is mainly controlled by two sets of genes: glaucousness loci (*W1* and *W2*) and non-glaucousness loci (*Iw1* and *Iw2*). The non-glaucousness (*Iw*) loci act as inhibitors of the glaucousness loci (*W*). High-resolution comparative genetic linkage maps of the wax inhibitors *Iw1* originating from *Triticum dicoccoides*, and *Iw2* from *Aegilops tauschii* were developed by comparative genomics analyses of *Brachypodium*, sorghum and rice genomic sequences corresponding to the syntenic regions of the *Iw* loci in wheat. Eleven *Iw1* and eight *Iw2* linked EST markers were developed and mapped to linkage maps on the distal regions of chromosomes 2BS and 2DS, respectively. The *Iw1* locus mapped within a 0.96 cM interval flanked by the BE498358 and CA499581 EST markers that are collinear with 122 kb, 202 kb, and 466 kb genomic regions in the *Brachypodium* 5S chromosome, the sorghum 6S chromosome and the rice 4S chromosome, respectively. The *Iw2* locus was located in a 4.1 to 5.4-cM interval in chromosome 2DS that is flanked by the CJ886319 and CJ519831 EST markers, and this region is collinear with a 2.3 cM region spanning the *Iw1* locus on chromosome 2BS. Both *Iw1* and *Iw2* co-segregated with the BF474014 and CJ876545 EST markers, indicating they are most likely orthologs on 2BS and 2DS. These high-resolution maps can serve as a framework for chromosome landing, physical mapping and map-based cloning of the wax inhibitors in wheat.

## Introduction

The outermost wax layer protects plants from many types of biotic and abiotic stresses, such as drought, phytophagous insects, pathogens, solar radiation, and freezing temperatures [1], [2]. One of the most important roles of the cuticle is to limit transpiration to reduce water loss and this provides a key mechanism for plant survival in water-limited environments, such as deserts, high mountains, saline-alkali lands, and coastal ecosystems [3], [4]. Worldwide, bread wheat (*Triticum aestivum* L.) is one of the most important food sources for human beings. The wheat leaf, stem and, in some cases, spike surfaces are coated with cuticular waxes that confer a glaucousness characteristic [5], [6]. Physiological studies in wheat by Johnson et al. [7] and Richards et al. [8] showed that glaucousness reduces transpiration and increased water use efficiency. More recently Zhang et al. demonstrated that glaucousness reduced cuticle permeability in the terms of non-stomatal water loss and chlorophyll efflux [9]. Bread wheat cultivars with non-glaucousness traits exhibit significant yield increases with reduced solar radiation losses that enable continued photosynthesis during the grain filling period [10], and the trait may also provide resistance to aphids [11].

Glaucousness and non-glaucousness are parallel variations in wheat and its relatives. Classical genetic studies have shown that both the glaucousness and the non-glaucousness stem and leaf phenotypes are controlled by two sets of loci; the wax production genes *W1* and *W2* and the wax inhibitor genes *Iw1* and *Iw2*, respectively. The *Iw1* and *Iw2* non-glaucousness loci function as inhibitors of the *W1* and *W2* glaucousness loci, and could also inhibit other wax production genes in the wax pathway [5], [12], [13]. Genetic analyses have indicated that the *W1* wax production gene and the *Iw1* wax inhibition gene are located on chromosome 2BS with a genetic distance of 2 cM [12]. However, *W2* and *Iw2* are separated on chromosome 2DS where the *W2* locus is close to the centromere [12]–[15]. Two loci, *Iw3* and *Ws*, were also reported conditioning wax on spikes in wheat. Non-glaucousness locus *Iw3* was mapped on chromosome 1BS [16] and the *Ws* gene on the short arm of chromosome 1AS is responsible for glaucous spikes [17]. In addition to these genes, a major QTL (*QW.aww-3A*) that accounts for up to 52 percent of the flag leaf glaucousness variation has been detected in a doubled-haploid (DH) population [18].

Molecular mapping and cloning of genes controlling epicuticular wax in wheat is of great interests for understanding interactions between none-glaucousness genes (*Iw*) and glaucousness genes (*W*), as well as their effects on yield, and biotic and abiotic stresses. The *Iw1* locus originating in wild emmer is closely linked to the *Xcdo456* RFLP marker at the end of chromosome arm 2BS [19]. Liu et al. found that the *Iw1* locus is 18.77 cM away from the powdery mildew resistance gene *MlIW170* on chromosome 2BS [20]. Simmonds et al. also reported that the *Iw1* (*Vir*) gene conditioning a non-glaucousness phenotype maps to chromosome 2BS [10]. In a tetraploid wheat background, Yoshiya et al., have found that *W1* is linked to *Iw1^Dic^*, but the relationship between *Iw1* and *Iw1^Dic^* was not confirmed [21], and in an *Ae. tauschii* F_2_ segregating population, the non-glaucous locus *Iw2* was located on chromosome 2DS [22]. In another report, the dominant non-glaucous locus *Iw3672* (*Iw2*) derived from a synthetic hexaploid wheat also mapped on 2DS by simple sequence repeat (SSR) and expressed sequence tag (EST) markers [23]. During development of a wheat genetic linkage map with a doubled haploid (DH) population derived from the TA4152–60 synthetic hexaploid wheat line and the ND495 common wheat line, Chu et al., also located a dominant wax inhibitor *Iw2* on chromosome 2DS [24]. Compared to studies on the *Iw* non-glaucousness loci, little work has been done to map the *W* glaucousness loci in wheat, aside from *W1*, which has been mapped on chromosome 2BS [21] and the *Ws* glaucous spike allele that is located at the terminus of chromosome 1AS [17].

The development of a high-resolution genetic linkage map is essential for fine mapping and map-based cloning of genes of interest. However, it is a tedious undertaking to develop refined genetic maps and to clone genes from wheat due to the huge genome size (16,000 Mb), polyploidy, high content of repetitive DNA and un-availability of a reference genome sequence. However, since several grass species including *Brachypodium distachyon* L., rice (*Oryza sativa* L.), sorghum (*Sorghum bicolor* L.), and maize (*Zea mays* L.) have been sequenced, and genome information from these species provides important resources for comparative genomics approaches and for development of high-resolution genetic linkage maps of genes of interest in wheat [25]. Successful examples of such approaches have been documented during cloning of the wheat vernalization gene *Vrn1* [26], the earliness *per se* gene *Eps-A^m^1* [27], and the durable leaf rust resistance gene *Lr34* [28]. Recently, the shotgun genome sequences of hexaploid wheat Chinese Spring [29], *Triticum urartu* [30] and *Aegilops tauschii* [31] provide more information for marker development to the genes interested in wheat.

In the present paper we have reported (1) identification and genetic analysis of the *Iw1* and *Iw2* wax inhibition genes originating from wild emmer and synthetic hexaploid wheat and (2) development of high-resolution comparative genetic linkage maps of *Iw1* and *Iw2* on chromosomes 2BS and 2DS, respectively.

## Materials and Methods

### Plant materials

Three mapping populations were selected for mapping of the wheat wax inhibition genes *Iw1* and *Iw2*. To map the *Iw1* locus, WE74, a non-glaucousness common wheat line derived from common wheat (glaucousness) and wild emmer (non-glaucousness) was used in crosses with Xuezao, a glaucousness common wheat line. These crosses produced a 4949 plant F_2_ segregating population and each F_2_ plant was bagged to harvest seeds for F_3_ family genotyping. A 120 line DH population developed from a hybrid between the non-glaucousness TA4152–60 synthetic hexaploid wheat line and ND495, a glaucousness common wheat line, was used to map the *Iw2* locus [24]. The newly developed International Triticea Mapping Initiative (ITMI) reference mapping population consisting of 1161 recombinant inbred lines (RIL) also was selected for mapping of the *Iw2* locus [32]. The glaucousness trait was phenotyped on each F_2_ plant, F_3_ family, RILs, and DH lines in field trials with adult plants. Chromosomal arm assignment and bin mapping of markers linked to the wax inhibition genes *Iw1* and *Iw2* were carried out with Chinese Spring (CS) and homoeologous group 2 nullisomic-tetrasomics [33], ditelosomics [34] and deletion lines [35].

### PCR and product analysis

Total genomic DNA was isolated from leaves by use of a cetyl trimethylammonium bromide (CTAB) protocol [36]. Non-glaucous and glaucous bulks, assembled with equal amounts of DNA from 10 homozygous non-glaucous and 10 homozygous glaucous F_2_ plants, were used for bulked segregant analysis (BSA) [37]. SSR and EST markers located on the short arms of homoeologous group 2 chromosomes were chosen for polymorphism screening [38,39, http://wheat.pw.usda.gov/cgi-bin/graingenes/browse.cgi?class=marker]. Primers for EST markers were designed from EST sequences derived from the public NCBI EST database. The primer-designed criteria included a Tm of 50–65°C with no greater than a 3°C difference between primer pairs. Primer sequences and information about the bin-mapped EST markers are available at the GrainGenes database (http://wheat.pw.usda.gov). Polymorphic markers between the parental wheat lines, as well as glaucous and non-glaucous bulks, were genotyped on each F_2_ plant, RILs, and DH lines.

Polymerase chain reaction (PCR) was performed in 10 µl reactions containing 10 mM Tris-HCl, pH 8.3, 50 mM KCl, 1.5 mM MgCl_2_, 0.2 mM dNTPs, 25 ng of each primer, 50 ng of genomic DNA, and 0.75 U of *Taq* DNA polymerase. Amplification of DNA was conducted at 94°C for 5 min, followed by 40 cycles at 94°C for 45 s, 50–60°C (depending on specific primers) for 45 s, and 72°C for 90 s, and reactions were terminated after a final extension at 72°C for 10 min. PCR products were mixed with 2 µl of loading buffer (98% formamide, 10 mM EDTA, 0.25% bromophenol blue, and 0.25% xylene cyanol), separated on 8% non-denaturing polyacrylamide gels (39 acrylamide ∶ 1 bisacrylamide), and visualized following silver staining.

### Comparative genomics analysis and EST marker development

Polymorphic bin-mapped EST markers flanking the non-glaucousness loci *Iw1* and *Iw2* were used in BLAST searches of the *Brachypodium*, sorghum, and rice genome sequences to find orthologous genomic regions. Orthologous gene pairs in the corresponding genomic regions between the three species were located and used to search homologous wheat ESTs (http://blast.ncbi.nlm.nih.gov/Blast.cgi) that were used to design PCR primers using Primer5.0 (http://www.genome.wi.mit.edu/ftp/pub/software/primer5.0). Polymorphic EST markers between non-glaucous and glaucous parental lines, as well as the bulk segregants, were used for genotyping three mapping populations to construct high-resolution genetic linkage maps.

### Data analysis and genetic linkage map construction

Chi-squared (χ^2^) tests for goodness-of-fit were performed to estimate deviations of observed data from theoretically expected segregation ratios. Linkages between molecular markers and the wax inhibition loci were analyzed using Mapmaker 3.0 with a LOD score threshold of 3.0 [40]. The genetic linkage map was drawn with the software Mapdraw V2.1 [41].

## Results

### Genetic analyses of wax inhibitors *Iw1* and *Iw2* in hexaploid wheat

At the adult plant stage, leaves and stems of the common wheat line, WE74, and the TA4152–60 and W7984 synthetic hexaploid wheat lines were non-glaucous, whereas the common wheat lines, Xuezao, ND495, and Opata M85, were glaucous. F_1_ plants from the Xuezao/WE74, ND495/TA4152–60, and W7984/Opata M85 crosses were non-glaucous, suggesting that the wax inhibitor genes in WE74, TA4152–60 and W7984 are dominant. The F_2_ population of Xuezao/WE74 segregated as 3730 non-glaucousness and 1219 glaucousness, which fits a 3∶1 ratio (Table 1). The F_2∶3_ progenies segregated as 1269 homozygous non-glaucousness ∶ 2461 segregating ∶ 1219 homozygous glaucousness, as expected for a single gene segregation ratio of 1∶2∶1 (Table 1). The ND495/ TA4152–60 DH lines segregated identical as reported by Chu et al. [22]. The 1161 RILs of the ITMI population segregated as 549 non-glaucousness and 612 glaucousness to fit the expected 1∶1 ratio (Table 1). These results indicate that non-glaucousness in the WE74, TA4152–60, and W7984 wheat lines is controlled by single dominant wax inhibitor gene. Since non-glaucousness originates from wild emmer in WE74 and synthetic wheat lines in TA4152–60 and W7984, the non-glaucousness loci should be designated *Iw1* in WE74 and *Iw2* in TA4152–60 and W7984, respectively.

**Table 1:** Genetic analysis of wax inhibitors *Iw1* and *Iw2* in hexaploid wheat.

Mapping population	Non-glaucousness^*^	Glaucousness^*^	Total	χ^2^	χ^2^_0.05_
WE74 (*Iw1*)	10				
Xuezao		10			
Xuezao/WE74	10				
Xuezao/WE74 F_2_	3730	1219	4949	0.36	3.84
Xuezao/WE74 F_3_	1269(A)+2461(H)	1219(B)	4949	1.16	5.99
W7984 (*Iw2*)	27				
Opata M85		20			
W7984/Opata M85 F_1_	26				
W7984/Opata M85 RILs	549	612	1161	3.41	3.84

A, H, B represent homozygous non-glaucousness, heterozygous and homozygous glaucousness, respectively.

### Identification of SSR markers linked to *Iw1*

**Figure 1:**
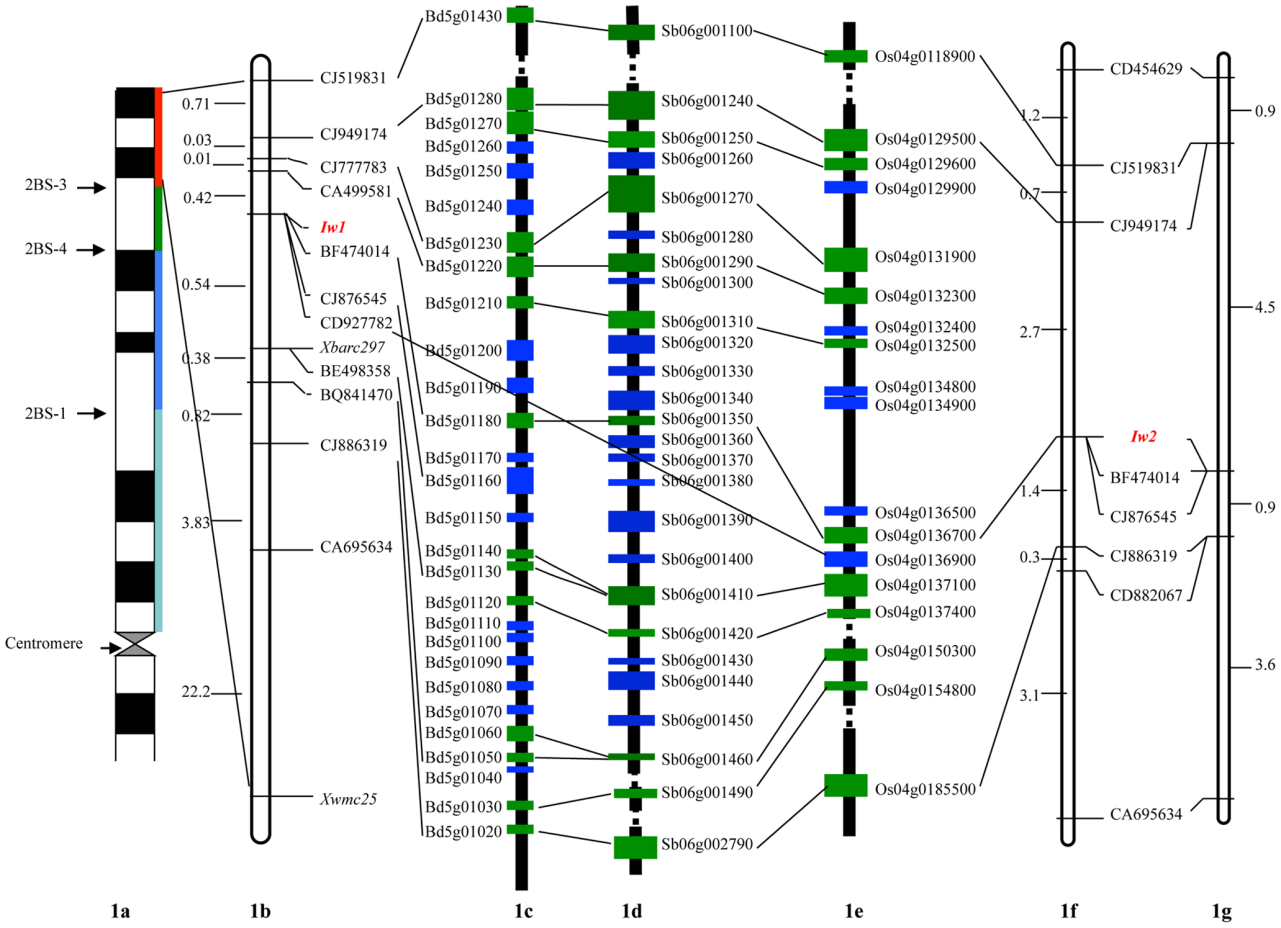
Comparative high-resolution genetic linkage maps of the wax inhibitors *Iw1* and *Iw2* in wheat. (1a) Physical bin map of *Iw1*. *Iw1* was mapped to distal bin 2BS3-0.84-1.00. (1b) Genetic linkage map of *Iw1* on wheat chromosome 2BS with genetic distances in cM shown on the left, markers shown on the right. (1c) The orthologous genomic region of *Iw1* on *Brachypodium* chromosome 5 with putative genes on the left. (1d) The orthologous genomic region of *Iw1* on sorghum chromosome 6 with putative genes on the right. (1e) The orthologous genomic region of *Iw1* on rice chromosome 4 with putative genes on the right. (1f) Genetic linkage map of *Iw2* on wheat chromosome 2DS using the ITMI population. Genetic distances in cM are shown on the left, and markers are shown on the right. (1g) DH population genetic linkage map of *Iw2* on wheat chromosome 2DS. The markers are shown on the left, and genetic distances in cM are shown on the right. The lines and green solid boxes indicated orthologous gene pairs and the blue solid boxes indicated non-orthologous genes between wheat, *Brachypodium*, rice and sorghum.

Because the *Iw1* and *Iw2* non-glaucousness genes are located on chromosomes 2BS and 2DS, respectively [10], [12], [19]–[24], SSR markers assigned to 2BS and 2DS were used preferentially for BSA. Linkage of the *Xbarc297* and *Xwmc25* polymorphic SSR markers to *Iw1* on chromosome 2BS was confirmed after genotyping the F_2_ segregating populations of the parental lines Xuezao and WE74, as well as the non-glaucous and glaucous DNA pools of Xuezao/WE74 (Fig. 1b). Two SSR markers *Xgwm614* and *Xgwm210*, previously linked to *Vir* or *Iw1* on chromosome 2BS [10], were not polymorphic between Xuezao and WE74, or the non-glaucous and glaucous DNA pools, and therefore could not be used for *Iw1* mapping.

### Physical bin map of *Iw1* and the linked SSR and EST markers

A set of Chinese Spring homoeologous group 2 nullisomic-tetrasomics, ditelosomics, and deletion lines [33]–[35] were employed for physical bin mapping of *Iw1*. The *Xwmc25* and *Xbarc297* SSR markers were located on chromosome 2BS bin 0.84–1.00 (Fig. 1a), indicating that the non-glaucousness locus *Iw1* was mapped on the distal part of 2BS.

The wheat ESTs physically mapping on chromosome 2BS bin 0.84–1.00 were screened for polymorphisms between Xuezao and WE74, as well as the non-glaucous and glaucous DNA pools. One EST marker, BE498358, was found to be linked to *Iw1* (Fig. 1b).

### Comparative analysis of the *Iw1* genomic region

The BE498358 EST sequence was used as a query to perform a Blast search against the *Brachypodium*, sorghum, and rice genome sequences. This search revealed that Bradi5g01130, Sb06g01410, and Os04g0136700 are orthologs of BE498358 located on the 5S, 6S, and 4S chromosomes of *Brachypodium*, sorghum, and rice respectively. Putative genes flanking Bradi5g01130, Sb06g01410, and Os04g0136700 were annotated and compared to identify orthologous gene pairs between the three species. The results indicate that a 462 kb genomic region in the *Brachypodium* chromosome 5S from Bradi5g01020 to Bradi5g01430 is syntenic to a 3.9 Mb region from Sb06g001110 to Sb06g002790 on sorghum 6S and a 5.6 Mb region from Os04g0118900 to Os04g0185100 on rice 4S (Table 2; Fig. 1). *Brachypodium* genes in the syntenic genomic region were then used to find homologous wheat ESTs to design primers and for polymorphism screening of the parental lines Xuezao and WE74, and the non-glaucous and glaucous DNA pools. Ten polymorphic EST markers were developed and used to genotype F_2_ individuals and to construct a high-resolution genetic linkage map of *Iw1* (Table 3; Fig. 1b). All 11 EST markers mapped to the *Iw1* genomic region, and 8 of these could be used simultaneously to find orthologous gene pairs in *Brachypodium*, sorghum, and rice syntenic genomic regions. The EST CJ876545 (which is an ortholog of *Brachypodium* gene Bradi5g01160), and ESTs CD927782 and CA695634 were excluded. However, the homologous CJ876545 sequences in sorghum and rice were not located on chromosomes 6S and 4S, but instead are located on chromosomes 10 (Sb10g005570) and 6 (Os06g0182500), respectively. CD927782 was developed from the rice gene Os04g0136900, but the homologous sequence of this gene could not be found in the *Brachypodium* and sorghum genomes. CA695634 was located 5.57 cM away from *Iw1*, and thus was not used for further analysis. The *Iw1* locus co-segregated with the BF474014, CJ876545, and CD927782 EST markers and is flanked by BE498358 and CA499581 in a 0.96 cM interval in the wheat genome that is collinear with a 122 kb genomic region with 10 predicted genes that encompasses Bradi5g01130 to Bradi5g01220 in *Brachypodium,* a 202 kb genomic region (Sb06g001290 to Sb06g001410) with 13 predicted genes in sorghum, and a 466 kb genomic region in rice (Os04g0132300 to Os04g0137100) that has 12 predicted genes (Table 2; Fig. 1).

**Table 2:** Colinearity between Brachypodium, sorghum and rice in the syntenic genomic region of wheat wax inhibitors *Iw1* and *Iw2.*

Wheat EST	Brachypodium	Sorghum	Rice	Annotation
CJ519831	Bradi5g01430	Sb06g001110	Os04g0118900	Hypothetical protein
CJ949174	Bradi5g01280	Sb06g001240	Os04g0129500	Sec24-like transport protein
	Bradi5g01270	Sb06g001250	Os04g0129600	Sec23/Sec24 zinc finger domain containing protein
	Bradi5g01260			Tyrosine specific protein phosphatase-like
	Bradi5g01250			Tyrosine specific protein phosphatase-like
	Bradi5g01240			Hypothetical protein transferase family protein
CJ777783	Bradi5g01230	Sb06g001270	Os04g0131900	UDP-glucose:sterol glucosyltransferase
CA499581	Bradi5g01220	Sb06g001290	Os04g0132300	AAR2 family protein
	Bradi5g01210	Sb06g001310	Os04g0132500	LRR receptor-like serine/threonine-protein
	Bradi5g01200			N-acylethanolamine amidohydrolase
	Bradi5g01190			Unknown protein/serine-type peptidase
BF474014	Bradi5g01180	Sb06g001350	Os04g0136700	CBS domain containing protein
CJ876545	Bradi5g01160			LIM, zinc-binding; Ubiquitin interacting motif; DA1-large seed size
	Bradi5g01150			Plant lipid transfer protein/protease inhibitor/seed storage
CD927782			Os04g0136900	Conserved hypothetical protein
	Bradi5g01140	Sb06g001410	Os04g0137100	Pectate lyase 15-like
BE498358	Bradi5g01130	Sb06g001410	Os04g0137100	Pectate lyase 15-like
	Bradi5g01120	Sb06g001420	Os04g0137400	Hypothetical protein
	Bradi5g01110			NB-ARC domain
	Bradi5g01100			NB-ARC domain
	Bradi5g01090			NB-ARC domain
	Bradi5g01080			NB-ARC domain
	Bradi5g01070			NB-ARC domain
	Bradi5g01060	Sb06g001460	Os04g0147200	Hypothetical protein
BQ841470	Bradi5g01050	Sb06g001460	Os04g0150300	Hypothetical protein
	Bradi5g01040			Hypothetical protein
	Bradi5g01030	Sb06g001490	Os04g0154800	Hypothetical protein
CJ886319	Bradi5g01020	Sb06g002790	Os04g0185500	Zinc finger family protein
CD882067	Bradi5g01010		Os04g0185100	CDT1-like protein a, chloroplastic-like

**Table 3:** EST markers of the wax inhibitors mapped in the *Iw1* and *Iw2* genomic regions.

Wheat EST	Forward sequence (5'–3')	Reverse sequence (5'–3')
CJ519831	ATACCAAGCCTACTAAGACACTG	AAGGCATACTCAACAGAAATCA
CJ949174	TGCTTGGGAATCTGTAATGC	GCTAACAAATCTGTGGACCTT
CJ777783	GCACTCGAAATGACTGGACA	CACTGCCTTACACTGCAGGA
CA499581	GTCACGCTCCTGCTCCTC	GCACCATCTTGATCCCTCTG
BF474014	CCAGTACCTCGAGTCCCAGA	CGAAGAGGGCGTCGATCT
CJ876545	CAAAATGTGATGTCTGCAAGC	CGATAAGGCCGTTCATATTTGT
CD927782	TCAGGCAACCAAAACCCTTA	CCTTTTCTCCAGCTCAATCG
BE498358	CAACTACTTCACCCACCACA	CACTGTGACCCAGGAGCATC
BQ841470	TTGTTCCGCCTGTATGATGA	GGAATCCTCATTGGACGAGA
CJ886319	GCCATCGGCGTAGTCTTC	TGGCTTGAAGCAGTGGAAGT
CD882067	GCGGCAGAAGCTCATATCAT	TGGGACAAACTCTAGCAGCA
CA695634	TTAGAAACGACAGTGCAGGG	GGTGCAAGTACAGAGGAGCC

### Comparative genetic mapping of *Iw1* and *Iw2*

EST markers linked to *Iw1* were used to genotype the 1161 ITMI RILs and 120 DH lines of ND495/TA4152–60 to develop a high-resolution genetic linkage map of *Iw2*. Eight EST markers linked to *Iw1* were polymorphic between ND495 and TA4152–60, as well as W7984 and Opata M85, and were used to construct a linkage map of *Iw2* on wheat chromosome 2DS (Fig. 1). The *Iw2* locus co-segregated with EST markers BF474014 and CJ876545, and is flanked by the ESTs CJ886319 and CJ519831 within a 4.1 cM sequence in the ITMI RIL population (Fig. 1f) and a 5.4 cM interval in the ND495/TA4152–60 DH population (Fig. 1g) on wheat chromosome 2DS which is collinear with a 2.3 cM genomic region spanning the *Iw1* locus on 2BS (Fig. 1).

## Discussion

The aerial surfaces of most plants are coated by epicuticular waxes whose chemical and physical properties have important roles in interactions between plants and the environment. In wheat and its relatives, almost all species have parallel variations of glaucousness and non-glaucousness except for Einkorn (A genome), which is non-glaucousness [6], [12], [14]. Genetic and cytological studies indicate that glaucousness is mainly controlled by two dominant genes, *W1* and *W2*, that are located on the distal of 2BS and proximal of 2DS, respectively; and are thought to be homologous [12], [13]. However, the glaucousness phenotype (controlled by *W1* and *W2*) is inhibited by the non-glaucousness *Iw1* and *Iw2* loci located on 2BS and 2DS, respectively [12], [14], [15]. These results indicate that the glaucousness locus (*W*) itself, and interactions between the non-glaucousness (*Iw*) and glaucousness (*W*) loci are responsible for wax phenotypes in different wheat tissues.

Cloning of wheat genes responsible for glaucousness and non-glaucousness will provide useful information about molecular interactions between the *W* and *Iw* loci, and the mechanisms whereby the waxy phenotypes are regulated. Our development of a high-resolution genetic linkage map is a first step towards fine mapping and map-based cloning of the glaucousness and non-glaucousness loci. However, additional refinements to the linkage maps are necessary before we can clone the respective genes and understand their relationships.

Comparative genomics analyses have been applied widely to develop high-resolution genetic linkage maps of interesting genes in wheat [25], [42], [43]. Macro-colinearity has been observed between wheat homoeologous group 2 chromosomes and *Brachypodium* chromosome 5, rice chromosome 4, and sorghum chromosome 6 [25], [44]–[46]. Several studies have also revealed high levels of micro-colinearity in particular genomic regions between wheat, *Ae. tauscii*, *Brachypodium*, and rice [20], [47]–[51] even through their synteny is often interrupted by inversions, deletions, duplications, and rearrangements [44], [46], [47], [49].

In this study, we have found that a 3.2 cM genomics region spanning the *Iw1* locus in wheat chromosome 2BS was highly syntenic to a 462 kb genomic region on *Brachypodium* chromosome 5S, a 3.9 Mb region on sorghum 6S, and a 5.6 Mb region on rice chromosome 4S (Fig. 1). However, gene duplications, insertions, and deletions were also observed in the syntenic genomic regions between wheat, *Brachypodium*, rice and sorghum. The *Iw1* co-segregating EST marker CJ876545 (orthologous to Bradi5g01160) is not found in the syntenic genomics regions of rice and sorghum, indicating that the *Brachypodium* gene order can serve as a better model than rice and sorghum for developing closely linked markers in wheat. However, another *Iw1* co-segregating EST marker, CD927782 (orthologous to Os4g0136900), was not located in the syntenic genomics region of *Brachypodium* and sorghum, implying that the rice and sorghum genes can provide alternative information for marker development of some wheat genes.

The bread wheat genome consists of three subgenomes (A, B, and D) that diverged from a common ancestor about 2.5–4.5 MYA [52], [53]. The three subgenomes are still very closely related after hundreds of thousands of years of independent evolution and genetic linkage maps and comparative analyses over the past 20 years have revealed substantial conservation of orthologs among the A, B, and D subgenomes [38], [39]. Conventional genetic analyses have also suggested that the *W1* and *W2* glaucousness loci are duplicated genes and that the *Iw1* and *Iw2* non-glaucousness loci are also duplicated [12], [13]. Additional molecular mapping experiments have revealed that both *Iw1* and *Iw2* are located on the distal part of chromosomes 2BS and 2DS, suggesting that they also may be orthologs. Low polymorphisms are observed on chromosome 2DS compared to chromosome 2BS, and of 11 EST derived markers mapping in the *Iw1* genomic region, only 7 are located on the *Iw2* genetic linkage map (Fig. 1). An F_2_ mapping population containing 4949 plants was used to narrow *Iw1* to a 0.96 cM genomic region flanked by the CA499581 and BE498358 EST markers and this region contained 10 predicted genes in the *Brachypodium* interval from Bradi5g01220 to Bradi5g01130, the same as the result of Adamski et al. [54]. However, by using 1161 ITMI RILs, *Iw2* could only be narrowed to a 4.1 to 5.4-cM genomic region corresponding to 26 predicted genes flanked by the CJ949174 and CJ886319 EST markers from Bradi5g01280 to Bradi5g01020 in *Brachypodium*. In common wheat, mapping of genes on the D chromosomes are often more difficult. The *Iw1* and *Iw2* also present such an example. Comparative genetic mapping results indicated that *Iw1* and *Iw2* are located in the orthologous genomic regions of chromosomes 2BS and 2DS and function as orthologs. The mapping results from *Iw1* will greatly help identification of *Iw2* genes. The EST markers BF474014 and CJ876545 are homologous to Bradi5g01180 and Bradi5g01160 and their co-segregation with *Iw1* and *Iw2* can serve as starting points for chromosome landing, physical mapping and map-based cloning of the non-glaucousness genes in wheat.
